# The prognostic role of WHO classification, urinary 5-hydroxyindoleacetic acid and liver function tests in metastatic neuroendocrine carcinomas of the gastroenteropancreatic tract

**DOI:** 10.1038/sj.bjc.6603699

**Published:** 2007-04-03

**Authors:** V Formica, A Wotherspoon, D Cunningham, A R Norman, B Sirohi, J Oates, G Chong

**Affiliations:** 1 Department of Medicine, Royal Marsden Hospital, London and Sutton, Surrey, UK; 2 Department of Histopathology, Royal Marsden Hospital, London and Sutton, Surrey, UK; 3 Department of Computing and Information, Royal Marsden Hospital, London and Sutton, Surrey, UK

**Keywords:** WHO classification, urinary 5-hydroxyindoleacetic acid, liver function tests, neuroendocrine carcinomas, gastroenteropancreatic tract

## Abstract

The World Health Organisation (WHO) classification (2000) is widely used to classify neuroendocrine carcinomas (NECs), yet its prognostic value needs to be confirmed. In this study, patients with metastatic NECs (*n*=119) were classified according to WHO guidelines into well differentiated and poorly differentiated (WDNECs and PDNECs). Histological differentiation based on WHO criteria had the highest impact on overall survival (OS) (PDNECs : WDNECs hazard ratio (HR)=4.02, *P*=0.02); however, PDNECs represented only a small percentage of patients (8%). In a WDNEC-restricted analysis, abnormal liver function tests (LFTs) and elevated urinary 5-hydroxyindoleacetic acid (u5HIAA) were independent prognostic factors for survival (HR=2.65, *P*=0.006 and HR=2.51, *P*=0.003, respectively) and were used to create a WDNEC-specific prognostic model (low risk=both normal, intermediate risk=one of them abnormal, high risk=both abnormal). Low-risk WDNECs had the most favourable prognosis (median OS, mOS 8.1 years), which was significantly better compared to both intermediate-risk and high-risk WDNECs (mOS 3.2 and 1.4 years, with *P*=0.01 and *P*<0.001, respectively). High-risk WDNECs displayed the shortest OS (1.3 years), which was similar to that of PDNECs (*P*=0.572). This analysis supports the prognostic value of WHO classification for metastatic NECs arising from the gastroenteropancreatic tract; however, risk stratification using readily available u5HIAA and LFTs may be necessary for the heterogeneous group of WDNECs.

Neuroendocrine tumours (NETs) are a group of tumours originating from cells of the diffuse endocrine system (DES). The DES includes a number of endocrine cells spread throughout all the body with the ability to secrete several bioactive amines and peptides. Intracellular structure is characterised by cytoplasmic electron-dense granules and immunohistochemistry shows the expression of antigens commonly found in nerve elements (e.g. chromogranin A (CgA), NSE, synaptophysin, PGP 9.5) (Rindi *et al*, 2000; Rindi and Kloppel, 2004). Diffuse endocrine system of gastroenteropancreatic (GEP) tract represents the largest portion with at least 15 different types of neuroendocrine cells identified. Most common NET, the carcinoid tumour, originates from enterochromaffin cells and characteristically secretes serotonin, tachykinins and other mediators responsible for the ‘carcinoid syndrome’ (for a review see Kulke and Mayer, 1999). Other hypersecretory syndromes (HSS) may be associated with NETs (e.g. insulinomas and gastrinomas) but in approximately 50% of cases they are ‘non-functioning’ (Plockinger *et al*, 2004).

In 2000, in consultation with the WHO, a revised classification for NETs was developed to unify histological diagnostic criteria and overcome the ambiguity of terms such as APUDoma, carcinoid and islet cell tumour (Solcia *et al*, 2000). The new WHO classification encourages the application of a unique scheme for all anatomical sites (lungs, thymus, gut and pancreas) and the fully comprehensive term of ‘neuroendocrine tumour’ (or ‘endocrine tumour’) over the above mentioned names (Rindi and Bordi, 2003; Rindi and Kloppel, 2004). Three main categories are identified: (1) well-differentiated neuroendocrine tumour (localised to the organ or bowel wall), (2) well-differentiated neuroendocrine carcinoma (WDNEC, locally advanced or metastatic) and (3) poorly differentiated neuroendocrine carcinoma (PDNEC, displaying high-grade histology regardless of tumour stage).

The classification provides a useful framework to study and classify NET patients, but its clinical relevance is not yet fully defined. The aim of this study was to assess the prognostic value of the WHO classification for metastatic NEC of the GEP tract and to evaluate whether other clinical, histological or biochemical parameters give additional prognostic information for this group of patients.

## PATIENTS AND METHODS

### Patients

Records of patients with a histological diagnosis of NET referred to the Royal Marsden Hospital (RMH) between April 1974 and May 2005 were retrospectively reviewed. Patients with metastatic disease arising from GEP tract were eligible for the study. Unknown primary site (UPS) tumours with intraabdominal metastases as the main site of disease were also included. Patients with a concomitant diagnosis of adenocarcinoma were excluded. All histological materials were reviewed by a single histopathologist (AW) and classified, according to the WHO classification, into well and poorly differentiated tumours. A distinction between carcinoid and non-carcinoid NETs was also made.

Baseline data including sex, age, tumour primary site, sites of metastases, performance status (PS), presence of HSS, routine haematology and biochemistry, 24 h level of urinary 5-hydroxyindoleacetic acid (u5HIAA) and other circulating tumour markers, uptake of radiotracer (radiolabelled metaiodobenzylguanidine and/or octreotide) and first treatment modality were recorded. Data were considered evaluable if collected within 3 months from the diagnosis and before any active treatment.

Patients were grouped by primary site according to the traditional classification of foregut, midgut and hindgut; a fourth group was formed by UPS tumours. Metastatic sites of disease were all grouped into nine categories (liver, pelvis, retroperitoneum, mesentery, peritoneum, mediastinum, lungs, bone, other). For patients diagnosed from 1994 onwards, staging included CT scan of the thorax abdomen and pelvis. However, for patients referred before 1994, chest radiograph and liver ultrasound were also considered acceptable for the purpose of this analysis. Four main HSS were recognised for functioning tumours: Carcinoid, Zollinger-Ellison, Cushing and Insulinoma-related syndrome. Performance status was measured using the Eastern Cooperative Oncology Group (ECOG) scale.

Biochemical parameters of liver function tests (LFTs) and u5HIAA were analysed. Liver function tests included bilirubin, alanine aminotransferase (ALT) and gamma-glutamyl-transferase (gammaGT) and an increase of grade 2 or more according to CTCAEv3.0 (Common Terminology Criteria for Adverse Events v3.0, http://ctep.cancer.gov/reporti ng/ctc.html) in at least one of the three LFT variables was required to define the tests as significantly abnormal. This was 2.5 times the upper limit of normal (ULN) for ALT and gammaGT and 1.5 times the ULN for bilirubin. Urinary 5-hydroxyindoleacetic acid was considered as the primary tumour marker for both carcinoid and non-carcinoid tumours; however, data from other markers (i.e. CgA, CEA, mucins, specific hormones) were also recorded when available. 24 h u5HIAA levels were defined as significantly elevated when higher than two times ULN. This cutoff was chosen because it corresponded to the median value. Data pertaining to first treatment modality were classified in five main categories: surgery, chemotherapy, biological therapy, radiotherapy and symptomatic therapy.

### Statistics

The primary statistical end point was overall survival (OS), defined as the interval time between diagnosis and death from any cause or last follow-up. The hazard ratios (HRs) between subgroups of patients with different features were estimated by Cox regression analysis, which was used for both univariate and multivariate analysis. Survival analysis was performed using the Kaplan–Meier method including confidence limits. The level of significance was 0.05 for all statistical tests. All analyses were performed using commercially available software (SPSS version 12, SPSS Inc., Chicago, IL, USA). Approval for the study was obtained from the local research ethics committee and committee for clinical research (study number 05/Q0801/167).

## RESULTS

### Patient characteristics

A total of 423 consecutive neuroendocrine patients were identified of whom 119 patients presenting with metastatic disease arising from the GEP tract at diagnosis were eligible for this analysis. Among them, 25 patients with an unknown primary site tumour (UPS tumours) were included as they had predominantly abdominal disease (usually liver and peritoneal surface) (Table 1).

The median age was 59 years (range 28–86 years); the male to female ratio was unity (1 : 1). The most common primary site was ileum (in 33% of patients), followed by pancreas (22%). According to the embryological classification of carcinoid tumours, midgut tumours were most common (43%). A high percentage of UPS NECs were observed (31%). The liver was involved as a site of metastasis in 72% of cases (37% liver-only, 35% with other organ metastases). After histopathological review, nine patients (8%) were classified as PDNEC and 110 (92%) as WDNEC.

At diagnosis, 38% of patients presented with symptoms and signs suggestive of HSS, in all of these the relevant hormone was elevated in the bloodstream. Performance status was available only for 55 patients (46%); it was therefore not included as variable in Cox-regression analysis. However, a PS of 3–4 was observed in only two patients.

Liver function tests and u5HIAA levels were not available for 12 and 19% of patients, respectively. Interestingly, for 10 patients with significantly elevated u5HIAA the histology was reported as showing a non-carcinoid tumour. The first therapeutic decision was debulking surgery for 45% of patients, chemotherapy for 26%, biological therapy (interferon and/or somatostatin analogues) for 13% and radiotherapy for 1%. Fifteen percent of patients were initially managed expectantly.

### Univariate analysis

Sex, age, primary site, sites of metastasis, histological differentiation, presence of HSS, LFTs and u5HIAA levels were considered as potential prognostic variables in the univariate analysis for OS (Table 2). Histological differentiation displayed the highest impact on prognosis, with an HR (PDNEC/WDNEC) of 3.37 (95% CI 1.50–7.55, *P*=0.003). Relative risk of death was also significantly higher for patients with abnormal u5HIAA or LFTs (HR 1.87, 95% CI 1.08–3.24, *P*< 0.025 and HR 2.08, 95% CI 1.17–3.72, *P*< 0.013, respectively), whereas midgut NETs were associated with a favourable prognosis when compared to the entire group of ‘non-midgut’ tumours (HR 1.74, 95% CI 1.08–2.81, *P*< 0.023). Relative risk of death was not significantly affected by sex, age, carcinoid nature of the tumours and presence of HSS.

### Multivariate analysis

Prognostic factors found to be statistically significant at univariate analysis were selected for multivariate analysis (Table 2). Histological differentiation was confirmed to be the most powerful independent prognostic factor, with a risk of death nearly four times higher when PDNEC was diagnosed (HR 4.02, 95% CI 1.26–12.82, *P*=0.02). Elevated u5HIAA levels and abnormal LFTs were also demonstrated to be independently associated with a poor outcome (HR 2.36, 95% CI 1.28–4.35, *P*=0.006 and HR 2.21, 95% CI 1.12–4.34, *P*=0.02, respectively). Midgut location of tumours was not confirmed to be an independent prognostic factor.

### Survival analysis

**Figure 1:**
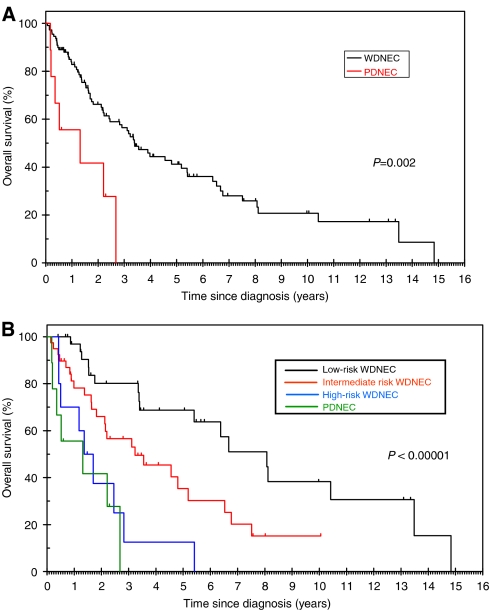
(**A**) Overall survival according to histological differentiation. WDNEC: well-differentiated neuroendocrine carcinoma; PDNEC: poorly differentiated neuroendocrine carcinoma. (**B**) Overall survival according to histological differentiation and the three risk groups for WDNEC; low-risk WDNEC: patients with both LFTs and u5HIAA not significantly altered; intermediate-risk WDNEC: patients with either LFTs or u5HIAA significantly altered; high-risk WDNEC*:* patients with both LFTs and u5HIAA significantly altered.

At the end of follow-up, 72 patients had died, 47 were alive (censored patients). Median OS (mOS) of the entire group was 3.2 years (95% CI 2.5–3.9) with a 2-year survival rate (2-y-OS) of 64.3% (95% CI 73–52%). Median follow-up was 2.3 years (95% CI 0.4–13.4). Overall survival was significantly shorter for PDNECs, with a mOS of 1.3 years and a 2-y-OS of 41.7%, compared to 3.4 years and 66.2%, respectively, for WDNECs, *P*=0.002 (Figure 1A).

### Risk model for WDNCs

A subanalysis restricted to WDNEC was performed to identify subgroups of these patients with different prognoses. The multivariate analysis confirmed LFTs and u5HIAA to be the most statistically significant prognostic factors (HR=2.65, 95% CI 1.33–5.30, *P*=0.006 and HR=2.51, 95% CI 1.35–4.63, *P*=0.003, respectively) (Table 3).

Liver function tests and u5HIAA were used to generate a WDNEC-specific prognostic model. The model identified three main classes of patients: (1) low-risk WDNECs=patients with both LFTs and 5HIAA not significantly altered; (2) intermediate-risk WDNECs=patients with one of the two variables significantly altered; and (3) high-risk WDNECs=patients with both LFTs and 5HIAA significantly altered.

A survival analysis was performed with both Kaplan–Meier and Cox-regression methods comparing the three WDNEC risk classes with PDNECs. Poorly differentiated neuroendocrine carcinomas displayed the shortest OS (mOS=1.3 years, 95% CI 0.1–2.8), but this was close to the survival of high-risk WDNEC patients (mOS=1.4 years, 95% CI 0.7–2.0) (Figure 1B). There was no significant difference between these two groups at Cox-regression analysis (HR=1.33, 95% CI 0.49–3.62, *P*=0.572) (Table 4). Low-risk WDNECs showed the most favourable prognosis (mOS 8.1 years, 95% CI 5.3–10.9), with a statistically significant difference when compared to both intermediate risk (mOS 3.2 years, 95% CI 1.4–5.1) and high risk (see above) (Figure 1B). On Cox-regression analysis, using low-risk WDNECs as the reference, HRs and *P*-values were HR=2.44, *P*=0.01 and HR=6.07, *P*=0.0001 for intermediate-risk WDNECs and high-risk WDNECs, respectively (Table 4).

## DISCUSSION

This study is the first reported single-centre data set selecting patients with metastatic GEP NET at diagnosis. Our retrospective analysis confirms the prognostic utility of histological differentiation according to the WHO classification. In addition, significant elevation of u5HIAA and/or LFTs predicts for decreased survival for patients with WDNEC.

Five-year OS for patients with metastatic NETs has been reported ranging from 26 to 73% in previous large series, showing a rather variable survival that needs to be further stratified (Eriksson *et al*, 1990; Madeira *et al*, 1998; Hochwald *et al*, 2002; Modlin *et al*, 2003).

The impact of histological differentiation on prognosis has been previously well documented. In a study by Madeira *et al* (1998) including NETs from the duodenopancreatic area and excluding carcinoids, PDNECs in patients presenting with liver metastases were associated with an HR for death of 8.11 (multivariate analysis). The rates of WDNECs and PDNECs were comparable to those observed in our study (WDNECs 78%, PDNECs 11%, non-evaluated 11%). In another study including only NETs of the stomach, mOS for PDNECs was 8 months (Rindi *et al*, 1999). In our analysis, histological differentiation was confirmed to be an independent prognostic factor, with the highest impact on OS. Poorly differentiated neuroendocrine carcinomas were associated with an mOS almost 2.5 times shorter than that observed for WDNECs (1.3 *vs* 3.4 years, *P*=0.002). Multivariate analysis showed a relative risk of death four times higher (*P*=0.02).

Poorly differentiated neuroendocrine carcinomas, however, were confirmed to represent only a small percentage of cases (8%), and on the remaining group (92% of patients) other discriminatory factors had to be applied. Univariate analysis showed u5HIAA level, site of origin (based on the distinction between midgut and non-midgut tumours) and LFT values to be significantly associated with prognosis, but only u5HIAA and LFTs were independent prognostic factors in the multivariate analysis (HR 2.4, *P*=0.006 and HR 2.2 *P*=0.02, respectively).

Urinary 5-hydroxyindoleacetic acid level was observed to be widely variable in WDNECs and, interestingly 10 patients not diagnosed as carcinoid tumours presented with significantly raised u5HIAA at diagnosis. Although it is well known that a given type of NET may secrete several bioactive hormones, the urinary excretion of 5HIAA in non-carcinoid tumours has rarely been documented. In a study by Meijer *et al* (2000) including a mixed population of healthy people, carcinoid NET patients, non-carcinoid NET patients and non-neuroendocrine cancer patients (688 subjects in total), 25 non-carcinoid NETs were associated with raised u5HIAA level and for 7 of these ‘high-levels’ of u5HIAA (higher than 2.4 times the ULN) were observed. Its prognostic influence, however, has been previously reported (Janson *et al*, 1997; Onaitis *et al*, 2000). An increasingly evaluated prognostic marker for NET, the tumour marker CgA, was not included in our analysis as it was not available for many patients (Baudin *et al*, 2001). Other studies including CgA are warranted.

Radiological degree of liver involvement in NETs is a well-documented prognostic factor (Janson *et al*, 1997; Madeira *et al*, 1998; Kouvaraki *et al*, 2004). Number of liver metastases (more than four) and proportion of liver volume involved (more than 75%) have been shown to correlate with poorer OS (Janson *et al*, 1997; Kouvaraki *et al*, 2004). We postulated that serum biochemical parameters such as gammaGT, ALT and bilirubin might provide prognostic information via a functional assessment of liver impairment. Our results showed that raised LFTs (⩾grade 2) are associated with a worse prognosis compared to patients with a grade 0–1 biochemical liver function. Radiological evidence of liver involvement alone was not significant for survival (*P*=0.960).

Contrary to what reported for localised NETs in previous studies, we could not demonstrate an impact of age and sex on prognosis (Janson *et al*, 1997; Hochwald *et al*, 2002).

From our analysis, it appears that functional liver impairment, as defined by elevation of LFTs ⩾grade 2, in combination with u5HIAA, can provide a simple and objective method for stratifying WDNEC patients in three prognostic classes. Patients were reasonably well distributed among the three classes (42, 46 and 12%, for LR, IR and HR-WDNECs, respectively) and survival differences were statistically significant. Looking at the Kaplan–Meier curves, high-risk WDNECs tend to have similar prognosis to PDNECs (mOS: 1.4 and 1.3 years, respectively), whereas low-risk and intermediate-risk WDNECs showed two distinct survival curves with an mOS of 8.1 and 3.2 years, respectively.

This single-centre series supports the prognostic value of the WHO classification for NETs. In addition, the analysis demonstrates that additional prognostic information may be gained by measuring u5HIAA and LFTs. Our proposed score may allow more accurate prognostic assessment within this heterogeneous patient population, although it needs to be confirmed in an independent data set. More importantly, improved risk stratification is critical for the development of future clinical trials. The identification of poor risk subgroups with WDNEC will hopefully assist in developing novel therapies for this relatively neglected group of patients.

## Figures and Tables

**Table 1:** Characteristics of 119 metastatic neuroendocrine tumour patients included in the analysis. Absolute number of patients (percentage within each group)

*Gender*		*Hypersecretory syndrome*	
Male	59	Functioning	45 (38%)
Female	60	Non-functioning	74 (62%)
*Age at diagnosis*		*Laboratory*	
Median	59 years	ALT	
Range	28–86	>2.5 × ULN	4
*Primary site*		< 2.5 × ULN	102
Foregut		NA	13
Pancreas	25 (22%)	GammaGT	
Stomach	1 (1%)	>2.5 × ULN	19
Midgut		< 2.5 × ULN	86
Ileum	40 (33%)	NA	14
Right colon	5 (4%)	Bilirubin	
Appendix	5 (4%)	>1.5 × ULN	6
Ceacum	3 (2%)	< 1.5 × ULN	100
Hindgut		NA	13
Left colon	3 (2%)	LFTs	
Rectum	1 (1%)	Raised	21
UPS GI	36 (31%)	Normal	84
*Site of metastasis*		NA	14
Liver	44 (37%)	u5HIAA	
Liver+other sites	42 (35%)	>2 × ULN	48
Other sites	33 (28%)	< 2 × ULN	48
*Differentiation*		NA	23
Well-differentiated	110 (92%)	*First clinical management*	
Poorly differentiated	9 (8%)	Symptomatic treatment	18 (15%)
*Carcinoid vs non-carcinoid*		Biological therapies	15 (13%)
Carcinoid	78 (66%)	Chemotherapy	31 (26%)
Non-carcinoid	41 (34%)	Surgery	53 (45%)
		Radiotherapy	2 (1%)

ALT=alanine aminotransferase; gammaGT=gamma-glutamyl transferase; LFTs=liver function tests; NA=not assessable; u5HIAA=urinary 5-hydroxyindole acetic acid; ULN=upper limit of normal; UPS GI=unknown primary site with abdominal lesions as main site of disease. LFTs raised: at least one of ALT, bilirubin or gammaGT abnormal.

**Table 2:** Univariate and multivariate analysis of factors prognostic for survival of the entire cohort

**Univariate analysis**					
**Variable**	**Group**	***N***	**HR**	**95% CI**	***P*-value**
Sex	Females	60	1		
	Males	59	0.73	0.45–1.17	0.190
Age (years)	<60	63	1		
	>60	56	1.04	0.65–1.66	0.862
Primary site	Hindgut	4	1		
	Foregut	26	0.22	0.07–0.70	0.01
	Midgut	53	0.16	0.05–0.48	0.001
	Unknown GI	36	0.30	0.10–0.89	0.029
Primary site	Midgut	53	1		
	Other sites	66	1.74	1.08–2.81	0.023
Metastases	Liver	44	1		
	Liver+other	42	1.45	0.83–2.53	0.19
	Other	33	0.98	0.54–1.80	0.96
Histology	Well-differentiated	110	1		
	Poorly differentiated	9	3.37	1.50–7.55	0.003
Histology	Non-carcinoid	41	1		
	Carcinoid	78	0.86	0.53–1.40	0.542
Functioning	No	74	1		
	Yes	45	1.18	0.73–1.91	0.5
LFTs	Non-signif. altered	84	1		
	Signif. altered	21	2.08	1.17–3.72	0.013
U5HIAA	Non-signif. altered	48	1		
	Signif. altered	48	1.87	1.08–3.24	0.025
**Multivariate analysis**					
Histology	Well-differentiated	84	1		
	Poorly differentiated	6	4.02	1.26–12.82	0.02
U5HIAA	Non-signif. altered	46	1		
	Signif. altered	44	2.36	1.28–4.35	0.006
LFTs	Non-signif. altered	73	1		
	Signif. altered	17	2.21	1.12–4.34	0.02
**Variables not significant in multivariate analysis**					
Primary site	Midgut	47			
	Other sites	43			0.06

*N*=absolute number of patients; CI=confidence interval; HR=hazard ratios; LFTs=Liver function tests; signif.=significantly; u5HIAA=urinary 5-hydroxyindole acetic acid; Unknown GI=unknown primary site with abdominal lesions as main site of disease.

**Table 3:** Multivariate analysis of factors prognostic for survival in selected patients with WDNEC

**Variable**	**Group**	***N***	**HR**	**95% CI**	***P*-value**
u5HIAA	Non-signif. altered	40	1		
	Signif. altered	44	2.51	1.35–4.63	0.003
LFTs	Non-signif. altered	69	1		
	Signif. altered	15	2.65	1.33–5.30	0.006
Primary site	Midgut	46	1		
	Other sites	38	1.86	1.04–3.31	0.03

CI=confidence interval; HR=hazard ratio; LFTs=liver function tests; *N*=absolute number of patients; signif.=significantly; u5HIAA, urinary 5-hydroxyindole acetic acid; WDNEC=well-differentiated neuroendocrine carcinoma.

**Table 4:** HRs and mOS for the three different WDNEC risk groups and PDNEC

**Group**	***N***	**mOS (years)**	**HR**	**95% CI**	***P*-value**	**HR**	**95% CI**	***P*-value**
Low-risk WDNEC	35	8.1	1					
Intermediate-risk WDNEC	39	3.2	2.44	1.24–4.80	0.01			
High-risk WDNEC	10	1.4	6.07	2.51–14.65	0.0001	1		
PDNEC	9	1.3	8.09	3.05–21.48	<0.0001	1.33	0.49–3.62	0.572

CI=confidence interval; HR=hazard ratio; LFTs=liver function tests; mOS=median overall survival; *N*=absolute number of patients; PDNEC=poorly differentiated neuroendocrine carcinoma; WDNEC=well-differentiated neuroendocrine carcinoma.

Low-risk WDNEC, patients with both LFTs and u5HIAA not significantly altered; intermediate-risk WDNEC, patients with either LFTs or u5HIAA significantly altered; high-risk WDNEC, patients with both LFTs and u5HIAA significantly altered.
